# Immortal Time-Bias–Corrected Survival of Highly Sensitized Patients and HLA-desensitized Kidney Transplant Recipients

**DOI:** 10.1016/j.ekir.2021.07.024

**Published:** 2021-08-02

**Authors:** Johan Noble, Antoine Metzger, Melanie Daligault, Eloi Chevallier, Mathilde Bugnazet, Beatrice Bardy, Hamza Naciri Bennani, Nicolas Terrier, Gaelle Fiard, Quentin Franquet, Benedicte Janbon, Dominique Masson, Diane Giovannini, Paolo Malvezzi, Thomas Jouve, Lionel Rostaing

**Affiliations:** 1 Nephrology, Hemodialysis, Apheresis and Kidney Transplantation Department, University Hospital Grenoble, Grenoble, France; 2 University Grenoble Alpes, Grenoble, France; 3 HLA Laboratory - Établissement Français du Sang (EFS), Grenoble, France; 4 Urology Department, University Hospital Grenoble, Grenoble, France; 5 Pathology Department, University Hospital Grenoble, Grenoble, France

**Keywords:** desensitization, end-stage renal disease, HLA-incompatible, kidney graft survival, kidney transplantation, patient survival

## Abstract

**Introduction:**

In the setting of kidney transplantation (KT), we assessed the efficacy of desensitization and compared the survival of desensitized patients (HLA-incompatible KT) with similarly sensitized patients receiving HLA-compatible KT or sensitized patients still on a waiting list after adjusting for the usually unaccounted immortal time bias.

**Methods:**

All patients in a French KT center on the waiting list between August 1994 and December 2019 with a high level of sensitization (panel-reactive antibodies [PRAs] ≥80%) were included. The primary outcome was all-cause mortality. A time-varying covariate Cox survival model was used to account for the immortal time bias. A landmark analysis was used as a sensitivity analysis.

**Results:**

During the study period, 326 patients with high PRAs were followed, among which 147 (45%) remained on the waiting list at the time of last follow-up and 179 benefited from a KT. Thirty-six patients were desensitized, of which 30 received a kidney transplant, including eight deceased kidney donors. There were no differences in mortality rates between desensitized KT patients, nondesensitized KT patients, and waitlisted patients after adjusting for immortal time bias (hazard ratio [HR] = 0.48, *P* = 0.22). Death-censored graft survival was similar between desensitized and nondesensitized KT patients (HR = 0.92, *P* = 0.88 adjusting for donor age >65 years, donor status, and time on the waiting list). Mean estimated glomerular filtration rate at 1 year post-KT was similar for desensitized KT patients (53.3 ± 21 vs. 53.6 ± 21 ml/min per 1.73 m^2^ for nondesensitized patients; *P* = 0.95).

**Conclusions:**

HLA-desensitization was effective for highly sensitized patients and gave access to KT without detrimental effects on patient or graft survival rates.

## Graphical abstract

**Figure:**
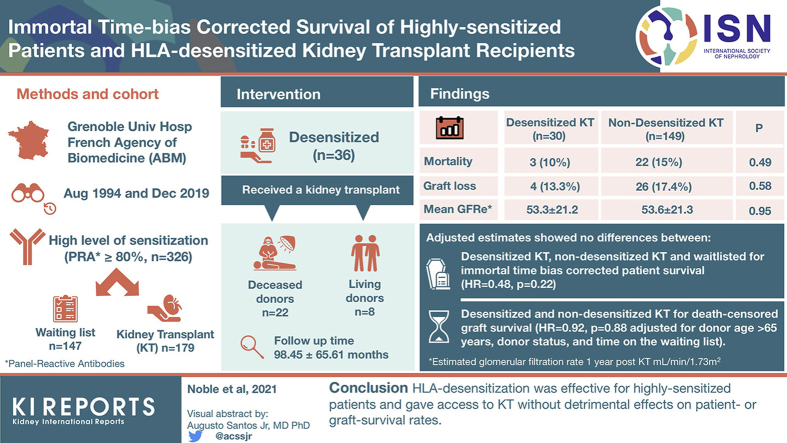

See Commentary on Page 2533

Chronic kidney disease and end-stage renal disease are global public health problems.^1^ KT provides the best results in terms of survival, quality of life, and health care savings compared to hemodialysis when kidney replacement is necessary.^2^ A major cause of restricting access to KT is a recipient’s anti-HLA sensitization. Highly sensitized patients are commonly defined by having a PRA percentage threshold of ≥80%. The PRA is calculated as the percentage of HLA antigens out of a panel reacting with the serum of a patient. It represents the percentage of donors expected to react with the serum of the patient.^3^

The number of sensitized and highly sensitized patients is increasing. In France, approximately 30% of patients on kidney transplant waiting lists are sensitized, of which 10% are highly sensitized. Highly sensitized patients remain on a waiting list for two to three times longer than nonsensitized candidates.^4^

Options for highly sensitized patients to receive a transplant are acceptable mismatch programs, paired donation, or desensitization.^5^ Desensitization significantly improves access to transplantation from deceased and living donors.^6^ Several studies report that HLA incompatible (HLAi) KT increases mortality and morbidity compared with compatible KT.^7^^,^^8^ Two large retrospective controlled studies have attempted to answer this question. In 2016, Orandi *et al.*^9^ showed a survival benefit in the United States for sensitized patients undergoing desensitization, followed by HLAi living-donor KTs, as compared to those remaining on the waiting list. However, the following year, Manook *et al.*^10^ found no significant survival advantage for desensitized patients compared to matched patients that remained on the waiting list in the United Kingdom. Moreover, the risk of rejection and graft failure after HLAi KT is still poorly defined.^11^

Thus, the issue of desensitization remains controversial: studies are poorly comparable due to heterogeneous desensitization protocols, baseline populations, hemodialysis care, and different matching methods.^12^, ^13^, ^14^ Furthermore, all studies focusing on the outcome of desensitized patients compared to wait-listed patients incurred an immortal time bias; that is, patients in the transplant group obviously have to survive at least until transplantation, forcing the survival time to be higher than in the control group (with a mean increase in time equal to the mean time between wait-listing and effective KT).^15^ The immortal time bias refers to the period when patients cannot experience the outcome during a period of the follow-up. By definition, KT patients did not die during the period between the inscription on waiting list and the transplantation. Otherwise they would have been included in the waiting list group of patients. This bias provides a statistical survival advantage to KT patients as compared to patients still on the waiting list. Neither the study by Orandi *et al.*^9^ nor the study by Manook *et al.*^10^ fully accounted for the immortal time bias. Finally, previous studies have included living donors only, preventing generalization to deceased donors KT.

In this retrospective study, we assessed the efficacy and safety of a homogenous desensitization protocol with respect to patient survival, rejection risk, and graft function in highly sensitized patients who underwent desensitization for HLAi KT and received a kidney from either a living or deceased donor. These patients were compared with similarly highly sensitized compatible (without desensitization) KT recipients, and with highly sensitized KT candidates who were still on the waiting list, while accounting for the immortal time bias.

## Materials and Methods

### Study Population and Definition

In this single-center study, all highly sensitized adult patients with a historical PRA ≥ 80% registered on the waiting list at Grenoble University Hospital between August 2, 1994, and December 31, 2019, were included. They were retrieved from the French database of candidates for adult KT managed by the French Agency of Biomedicine.

Patients who underwent desensitization for a living- or deceased-donor KT were referred to as "desensitized KT patients." Inclusion into the desensitization protocol required being on the KT waiting list, having no infectious or neoplastic comorbidities, and having optimal results of a cardiac check-up within the previous 3 months.

Control groups included patients with the same level of sensitization (PRA ≥ 80%) and that were on the KT waiting list. The two groups were defined according to patient status: those who received a kidney transplant without desensitization (“nondesensitized KT patients”) and those who did not receive a KT; that is, unlisted patients and patients still waiting for a transplant (“waiting list patients”) at the end of the follow-up period.

All medical data were collected from our database (CNIL [French National committee for data protection] approval number 1987785v0).

### Endpoints

The primary outcome was all-cause mortality. We assessed patient-survival rates since the inscription on kidney-waiting list for both desensitized KT and control groups (i.e., nondesensitized KT patients and those still on the waiting list or unlisted).

The secondary endpoints were graft survival, biopsy-proven acute rejection according to the most recent updated Banff classification, and graft function at last follow-up. We compared graft survival rates, biopsy-proven acute rejection incidence, and estimated glomerular filtration rate between the desensitized KT patients and nondesensitized KT patients. We also investigated the predictive factors for mortality in highly sensitized patients. A systematic graft biopsy was performed at 3 months.

### HLA antibodies

Screening for pretransplant HLA sensitization was performed using a bead assay (Luminex Single Antigen assay, Immucor, Norcross, GA). The first screening technique was followed by an ultrasensitive technique: the single-antigen technique. Screening and single antigen technique were performed for each patient on the KT waiting list before KT, during desensitization, on the day of transplantation, and then annually during the follow-up. The single-antigen technique was used to define mean fluorescence intensity (MFI). A donor-specific antigen (DSA) MFI > 3000 was considered defining unacceptable mismatch for all patients.

### Desensitization Procedure

Patients received two rituximab injections (375 mg/m^2^ each) at 30 and 15 days before living-donor HLAi KT or at the beginning of desensitization (days 5 and 12) for HLAi KT with a deceased donor. Immunosuppression therapy was started 10 days before a living KT or at the beginning of desensitization for a deceased-donor KT. The immunosuppressive regimen consisted of prednisone (0.5 mg/kg), mycophenolate mofetil (500 mg × 2 per day), and tacrolimus (initial dose 0.01 mg/kg per day, with a target trough concentration of between 8 and 10 ng/ml).

DSAs were monitored once a week. Apheresis sessions were performed by immunoadsorption, plasma exchange, or double-filtration plasmapheresis.

For living-donor KT, the protocol consisted of four or five apheresis sessions per week for 2 weeks before KT. If the DSA MFI was >12,000, immunoadsorption was performed daily. If DSA MFI was < 6000, immunoadsorption could be replaced by double-filtration plasmapheresis or plasma exchange to achieve a threshold MFI of < 3000 before KT. KT was performed when DSAs had an MFI of < 3000; that is, a negative-flow cytometric crossmatch in our center on the day before KT.

For deceased-donor KT, three to five apheresis sessions per week were performed until a compatible kidney graft was available. If no nationally available graft was proposed within 45 days after starting desensitization, the first local ABO-compatible graft matched for age and weight was proposed.

Patients with only historical DSA who received Rituximab without apheresis were not included in the desensitized group.

### Immunosuppression

All patients received 1 g of mycophenolate mofetil preoperatively, followed by mycophenolate mofetil 2 g/day, rapidly tapered to 1 g/day. Prednisolone is administered at the dose of 500 mg preoperatively, progressively tapered to 10 mg/day at day 30 post-KT. Tacrolimus is given and adjusted to achieve trough levels of 8 to 12 ng/ml. Induction therapy consisted of antithymocyte globulin for all patients (Genzyme, Lyon, France).

### Statistical Analyses

Quantitative data are presented as means ± SD or as medians with quartiles (interquartile range [IQR]). Qualitative data are presented as the numbers of patients and percentages. Missing data were removed for percentages calculation. The chi square test was used for categorical variables; the Wilcoxon or the Kruskal-Wallis test was used for continuous variables. Patient survival and KT survival were assessed using Kaplan-Meier curves and the log-rank test. Cox’s proportional hazards regression was used to assess the association between patient survival and clinical and biological characteristics. This multivariate model included all significant parameters in the univariate analysis.

The immortal time refers to a period during which death cannot occur. Herein, patients from the transplantation group could not die before transplantation (or their death would count as an event in the waiting list group).^16^

To account for this immortal time bias, we used a time-dependent Cox survival model, with the transplantation status as a time-varying covariate.^15^^,^^17^^,^^18^ In this model, for transplanted patients, time spent on the waiting list before KT was included in the waiting list group. The bias of mandatory survival up to the time of transplantation was therefore alleviated.

The landmark analysis is another method to avoid the immortal time bias by splitting the follow-up time at a common period for all groups. As a sensitivity analysis, we used a landmark analysis within a Cox's survival model.^15^^,^^19^ In this model, we set a landmark at 36 months post-registration on the waiting list because the minimal time on the waiting list to allow access to the desensitization program was 36 months and the median time spent on the waiting list was 33.8 months in our cohort. We finally used a multivariate version of this landmark analysis to evaluate other covariates (potential confounders) associated with survival.

A two-sided *P* value of < 0.05 was considered statistically significant. Statistical analyses were conducted using R statistical software.

## Results

### Study Populations

Between August 1994 and December 2019, 326 highly sensitized patients were wait-listed for a kidney transplant at Grenoble University Hospital. Among these, 36 participated in the desensitization protocol, 141 (43.2%) were still on the KT waiting list (or unlisted) at last follow-up, and 149 (45.7%) received a KT without desensitization at the time of inclusion (see flow chart in Supplementary Figure S1). Baseline characteristics of these patients are shown in Table 1. Data regarding age, sex, body mass index, and vascular impairment were similar between the three groups. Desensitized KT patients were less likely to be diabetic than patients on the waiting list or nondesensitized KT patients (*P* = 0.03). The median historical PRA was higher for desensitized KT patients (98%) versus nondesensitized patients and those on the waiting list (respectively, 94% and 97%; *P* < 0.001). Median follow-up of the study population from waiting list inscription until last follow-up was 83 (IQR: 53 to 127) months.

**Table 1:** Demographic characteristics at registration on waiting list for highly sensitized patients

	Transplanted w/o des (n = 149)	Transplanted with des (n = 30)	WL desensitized (failure) (n = 6)	WL only (n = 141)	Total (n = 326)	*P*
Recipient age,						0.05
mean (SD), years	50.07 (12.72)	46.75 (12.35)	41.28 (15.43)	52.31 (14.63)	50.57 (13.70)	
Female	90 (60)	16 (53)	3 (50)	73 (52)	182 (56)	0.50
Body mass index,						0.17
mean (SD), kg/m^2^	23.59 (4.96)	24.06 (4.05)	24.11 (6.86)	24.62 (4.93)	24.09 (4.91)	
HBP, n (%)	87 (75)	21 (84)	4 (80)	92 (77)	204 (77)	0.81
Vascular impairment^a^	15 (13)	6 (23)	1 (20)	27 (22)	49 (18)	0.24
Smoking history, n (%)	50 (45)	16 (67)	3 (60)	44 (40)	113 (45)	0.11
Diabetes	14 (11)	1 (4)	0 (0)	24 (20)	39 (14)	0.08
Follow-up time, months						<0.01
Mean (SD)	107.36 (48.95)	98.45 (65.61)	86.00 (62.90)	78.10 (55.98)	93.49 (55.50)	
Median (IQR)	105.69 (71.79 to 137.92)	92.50 (54.87 to 119.73)	86.44 (41.61 to 104.29)	64.89 (41.69 to 95.61)	83.04 (52.60 to 126.97)	
Historical PRA, %						<0.01
Mean (SD)	93.35 (4.80)	95.92 (4.39)	98.67 (2.34)	95.18 (4.35)	94.49 (4.66)	
Median (IQR)	94.00 (89.00 to 98.00)	98.00 (93.75 to 99.00)	99.50 (99.00 to 100.00)	97.00 (93.00 to 99.00)	95.50 (91.00 to 99.00)	

des, desensitization; HBP, high blood pressure; IQR, interquartile range; PRA, panel-reactive antigen; WL, waiting list; w/o, without.

Values are n (%) unless otherwise stated.

a Vascular impairment criteria include patients with a history of stroke, arteriopathy, or a coronary event.

#### Kidney Transplant Population

A total of 179 patients received a KT: their average age was 56 years. Of these, 133 (66.5%) had received at least one previous KT. The time on the waiting list before a KT for desensitized patients was 66 (IQR: 21 to 96) months, whereas it was significantly shorter in nondesensitized patients (i.e., 31 [IQR: 17 to 56] months; Wilcoxon *P* = 0.023). Similarly, and as expected, the time on the waiting list for living-donor transplantation was 13 (IQR: 8 to 43) months, significantly shorter than 36 (IQR: 20 to 71) months for deceased-donor transplantation (Wilcoxon *P* = 0.029). Regarding immunosuppressive regimen at transplantation, the two groups of KT patients were not comparable at baseline for the use of rituximab, mycophenolate mofetil, or mammalian target of rapamycin inhibitors (Table 2).

**Table 2:** Demographic characteristics of desensitized kidney transplant patients compared to nondesensitized kidney transplant patients

	Transplanted w/o des (n = 149)	Transplanted with des (n = 30)	Total (n = 179)	*P*
Living donors	6 (4)	8 (28)	14 (8)	<0.01
Donor age,				0.84
mean (SD), years	55.32 (13.86)	57.14 (11.51)	55.63 (13.48)	
Post-transplantation follow-up, months				<0.01
Mean (SD)	63.33 (39.29)	25.03 (17.41)	56.91 (39.23)	
Median (IQR)	62.49 (28.85 to 97.28)	25.35 (10.36 to 41.50)	50.66 (24.89 to 92.01)	
Transplantation rank,				
First kidney transplantation, %	49 (33)	10 (33)	59 (33)	0.97
ATG induction	147 (99)	29 (100)	176 (99)	0.53
Tacrolimus	149 (100)	29 (100)	178 (100)	
mTOR inhibitor	0 (0)	2 (7)	2 (1)	<0.01
MPA	149 (100)	25 (86)	174 (98)	<0.01
Rituximab	14 (9)	30 (100)	44 (25)	<0.01

ATG, antithymoglobulin; des, desensitization; HBP, high blood pressure; IQR, interquartile range; mTOR, mammalian target of rapamycin; MPA, mycophenolic acid; WL, waiting list; w/o, without.

Values are n (%) unless otherwise stated.

#### Desensitized KT Patients

Of the 36 desensitized patients, 30 (83%) received a transplant. Six desensitized KT patients did not receive a transplant because of failure to remove HLA antibodies (three patients) or the occurrence of complications (three patients) (i.e., myocardial infarction, pulmonary infection, and digestive perforation). One patient died during the desensitization protocol period from acute coronary syndrome. Median follow-up time post-KT in desensitized patients was 25.3 (IQR: 9.5 to 41.5) months versus 62.5 (IQR: 28.2 to 96.9) for nondesensitized patients (*P* < 0.001).

### Primary Endpoint: Patient Survival Rates

**Figure 1:**
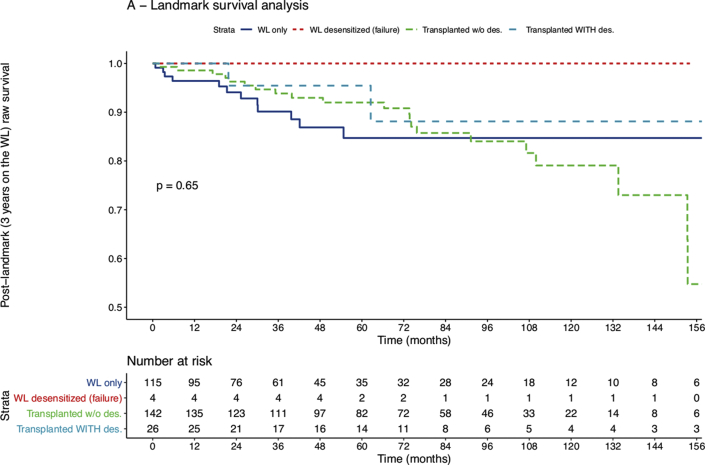
Landmark survival analysis within a Cox's model of desensitized patients and comparable highly sensitized patients. A landmark at 36 months postregistration on the waiting list was set. Survival curve of desensitized patients in the setting of HLA-incompatible kidney transplantation (light blue) is compared to highly sensitized patients remaining on the kidney-transplant wait-list (WL) at follow-up (dark blue), to highly sensitized patients that received a transplant without desensitization (green) and to desensitized patients who did not receive a kidney graft (red).

At last follow-up, among patients without a transplant, 33 were unlisted due to a degradation of their health status and 108 remained on the waiting list. Fourteen (42.4%) delisted patients died thereafter, 26 (24%) patients died on the waitlist, and 25 (13.9%) patients died after transplantation. In a time-dependent Cox's survival model, survival was not associated with transplantation (HR = 0.94, 95% confidence interval [CI] [0.50 to 1.76], *P* = 0.851) nor was desensitization (HR = 0.48, 95% CI [0.33 to 1.04], *P* = 0.222) in the multivariate model including recipient age, history of vascular impairment, body mass index, and PRA level. When comparing the modeled survival of the time-dependent Cox's model, survival was similar for wait-listed patients and for nondesensitized transplant patients, with a tendency towards better survival for desensitized transplant patients (Supplementary Figure S2). As a sensitivity analysis, we used a Cox's survival model with a 36-month landmark. In this model, raw survival was not associated with desensitization (HR = 0.43, 95% CI [0.1 to 1.79], *P* = 0.244) (Figure 1). With a median post-transplantation follow-up of 54 months, raw survival was also not impacted by transplantation itself (HR = 0.99, 95% CI [0.5 to 1.96], *P* = 0.982).

In a Cox multivariate model, to account for potential confounders, analysis adjusting for the covariates associated with survival in univariate analysis (Table 3), a history of vascular impairment was significantly associated with a worse survival (HR = 2.62, 95% CI [1.1 to 6.2]; *P* = 0.03), as well as an increased body mass index (HR = 1.08, 95% CI [1.01 to 1.16], *P* = 0.22). Desensitization was not associated with a worse graft survival in univariate analysis and therefore not included in this multivariate model (*P* = 0.58).

**Table 3:** Factors associated with patient death in univariate and multivariate analyses

	Univariate HR (95% CI)	Univariate *P* value	Multivariate HR (95% CI)	Multivariate *P* value
Recipient age, years	1.04 (1.01 to 1.07)	< 0.01	1.02 (0.99 to 1.06)	0.18
Donor age, years	1.01 (0.98 to 1.04)	0.53	—	—
High blood pressure	1.35 (0.51 to 3.59)	0.54	—	—
Diabetes	1.76 (0.67 to 4.66)	0.25	—	—
Vascular impairment	2.9 (1.25 to 6.71)	0.01	2.62 (1.1 to 6.2)	0.03
Body mass index, kg/m^2^	1.10 (1.05 to 1.17)	<0.01	1.08 (1.01 to 1.16)	0.02
Smoking history	1.38 (0.63 to 3.08)	0.43	—	—
Female	1.69 (0.87 to 3.29)	0.12	—	—
Desensitization	0.43 (0.1 to 1.77)	0.24	—	—
Historical PRA	0.91 (0.86 to 0.98)	0.01	0.95 (0.87 – 1.03)	0.18
Kidney transplantation	0.93 (0.47 to 1.82)	0.83	—	—
Rituximab use	0.34 (0.08 to 1.43)	0.14	—	—

CI, confidence interval; HR, hazard ratio.

Survival did not statistically differ between desensitized KT patients from living donors compared to desensitized KT from deceased donors (log-rank *P* = 0.19). Inclusion of donor status in the multivariate analysis did not change the results on survival.

Patient-survival rates at 1 year were 98% for nondesensitized KT and 97% for both wait-listed patients and desensitized KTs. After 3 years, patient survival rates were 95%, 92%, and 93%, respectively. Causes of death and graft loss in the desensitized group are detailed in Supplementary Table 1 (Table S1).

### Kidney Outcomes

#### Graft Survival

**Figure 2:**
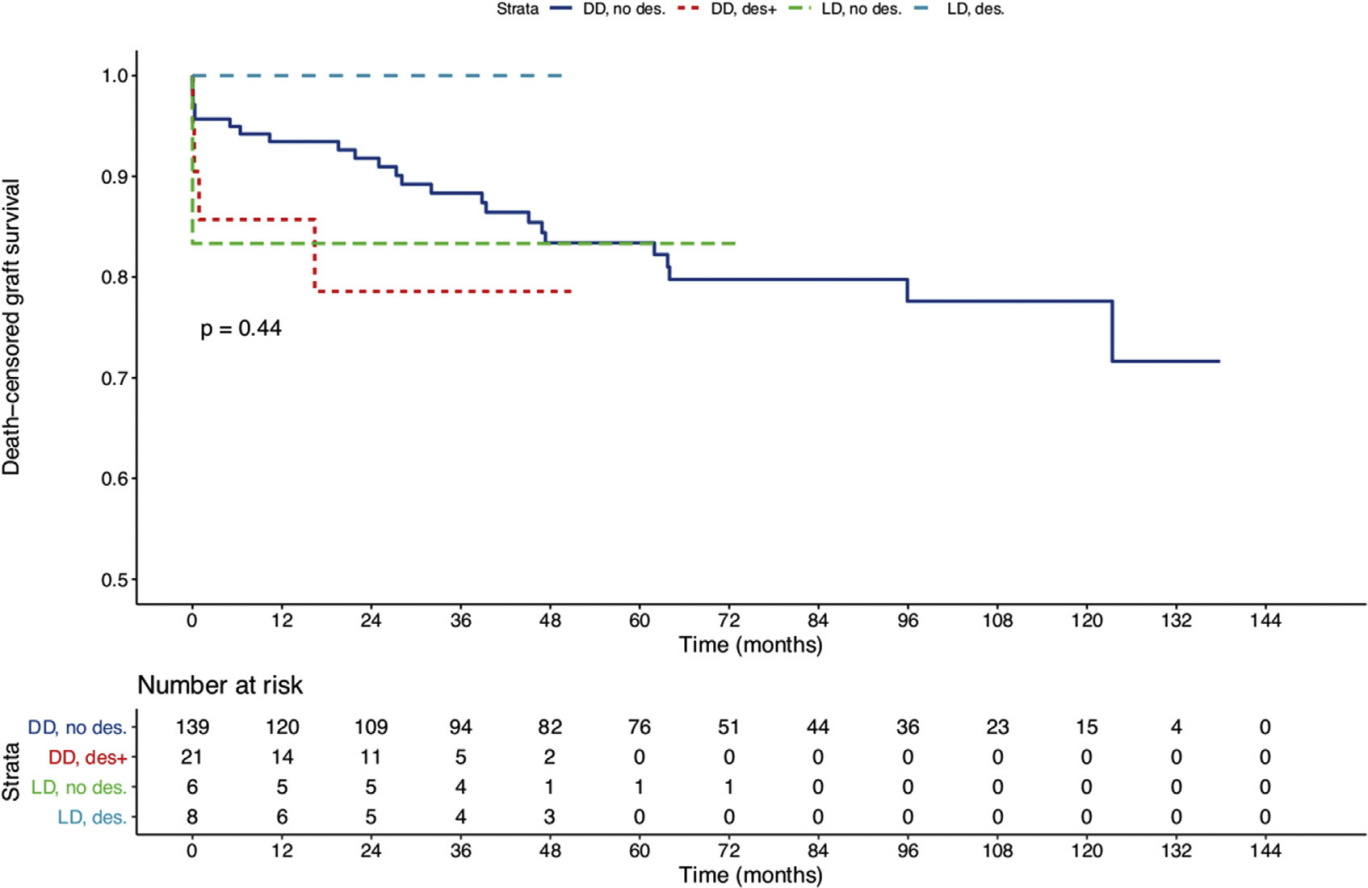
Overall comparison of kidney graft survival (death censored) between desensitized patients and patients that received a kidney transplant without desensitization. Kaplan-Meier estimates of death-censored kidney graft survival of desensitized patients in the setting of HLA-incompatible living donors (LD des, light blue), or deceased-donor (DD des, red) kidney transplantation to highly sensitized patients that received a living donor kidney transplant (LD no des, green), or deceased donors (DD no des, dark blue) without desensitization.

Death-censored graft survival (DCGS) did not differ between desensitized and nondesensitized KT patients (Figure 2) after a mean follow-up of 56.9 (IQR: 24.9 to 92) months post-KT (log-rank *P* = 0.63). In multivariate Cox's regression, adjusting for donor age, donor status (living vs. deceased), and time on the waiting list, DCGS was not associated with desensitization (HR = 0.92 [0.28 to 3.01]; *P* = 0.887) nor was donor age >65 years (HR = 1.14 [0.49 to 2.69]; *P* = 0.76), or donor status (HR = 0.66 [0.08 to 5.14]; *P* = 0.689). However, DCGS was associated with time spent on the waiting list (HR = 1.12 [1.03 to 1.2] per year spent on the waiting list; *P* = 0.005). At 1-year post-transplantation, graft survival rates were 96% for nondesensitized KT patients and 91.6% for desensitized KT patients (*P* = not significant). At 3 years, graft survival rates were 90.6% for nondesensitized KT patients and 88.8% for desensitized KT patients (*P* = not significant).

#### Rejection

The rate of T-cell–mediated rejection was similar between desensitized and nondesensitized recipients; at one (0.3%) and four (3.5%) cases, respectively (*P* = 1). However, in this population of highly sensitized patients, the number of antibody-mediated rejections (ABMRs) was greater in desensitized patients: 36.6% versus 9.6% in the nondesensitized patients (*P* < 0.001) (Table 4). Among the 11 ABMRs in the desensitized group, three did not receive treatment because they were asymptomatic and had minor glomerulitis (seen on allograft biopsy specimens). Regarding immunosuppressive regimen at last follow-up, there were no differences between the groups except for the use of steroids, which was more frequent in desensitized patients: 95% versus 39.5% in nondesensitized patients (*P* < 0.001).

**Table 4:** Kidney transplant parameters at last follow-up

	Transplanted w/o des (n = 149)	Transplanted with des (n = 30)	Total (n = 179)	*P*
Maintenance Tac	117 (91)	19 (95)	136 (91)	0.53
Tac trough levels, ng/ml				0.17
Mean (SD)	5.4 (1.5)	6.2 (3.0)	5.5 (1.8)	
Median (IQR)	5.1 (4.3 to 6.0)	5.8 (5.3 to 6.5)	5.3 (4.4 to 6.2)	
Maintenance MPA	103 (80)	20 (100)	123 (83)	0.03
MPA dose,				0.06
mean (SD), mg/day	856 (1340)	708 (180)	836 (1247)	
Maintenance steroids	51 (40)	19 (95)	70 (47)	<0.01
Steroid dose,				0.71
mean (SD), mg/day	5.3 (2.7)	5.5 (2.2)	5.3 (2.5)	
Delayed graft function	15 (12)	8 (27)	23 (15)	0.04
BPAR	20 (18)	12 (48)	32 (23)	<0.01
TCMR	4 (4)	1 (4)	5 (4)	0.90
ABMR	11 (10)	11 (44)	22 (16)	<0.01
Time to rejections, months				0.21
Mean (SD)	20.4 (23.4)	14.5 (16.5)	18.0 (20.7)	
Median (IQR)	9.3 (3.4 to 33.1)	6.0 (0.7 to 32.5)	9.1 (2.7 to 33.2)	
eGFR at 1 year post-KT, ml/min per 1.73 m^2^				
mean (SD)	53.6 (21.3)	53.3 (21.2)	53.5 (21)	0.95
Graft loss	26 (17.4)	4 (13.3)	30 (16.5)	0.58
Death^a^	22 (15)	3 (10)	25 (14)	0.49

Values are n (%) unless otherwise stated.

ABMR, antibody-mediated rejection, according to the Banff classification; BPAR, biopsy-proven graft rejection; des, desensitization; eGFR, estimated glomerular filtration rate, estimated according to the Modification of Diet in Renal Disease equation; IQR, interquartile range; KT, kidney transplantation; MPA, mycophenolic acid; Tac, tacrolimus; TCMR, T-cell–mediated rejection; w/o, without.

a The six desensitized patients that did not receive a kidney transplant are excluded from these analyses, except for the death criteria.

#### Kidney Function

Kidney graft function at 1 year post-transplantation was similar in desensitized KT patients compared to nondesensitized patients (estimated glomerular filtration rate: 53.3 ± 21 vs. 53.6 ± 21 ml/min per 1.73 m^2^, respectively; *P* = 0.95) (Table 4).

### DSA Trends of DSAs at Post-Desensitization and After KT

The mean predesensitization MFI of DSAs in desensitized KT patients was 7229 (IQR: 2470 to 13224). The mean predesensitization MFI of immunodominant anti-HLA antibodies in desensitized KT patients was 14,970 (IQR: 10,358 to 19,378). Immunodominant anti-HLA was anti–class I for 22 patients of 36 desensitized patients. The MFIs of DSAs were all <500 on the day of transplantation. At 1-year post-KT, DSA MFIs had a median of 261 (IQR: 0 to 627) for anti–class I DSAs and 743 (IQR: 0 to 463) for anti–class II DSAs.

## Discussion

In this study, overall survival was similar between desensitized patients, nondesensitized patients, and patients still waiting for a compatible KT. Moreover, graft survival was similar between desensitized patients and nondesensitized KT patients. Using a proper strategy to deal with the immortal time bias inherent to all studies that evaluate the impact of desensitization strategies on survival, we confirm that desensitization did not induce a higher death toll. We suggest a trend toward a better survival in desensitized patients. This is the first time this has been confirmed while accounting for the immortal time bias. Indeed, in the field of KT, the immortal time bias induces a survival advantage to KT patients, as compared to patients still on waiting list, because they *de facto* did not die during the period between the inscription and the transplantation. We believe that taking into account this bias is mandatory to compare the survival benefit of transplantation and desensitization and is the strength of this study.

In the study by Manook *et al.*,^10^ the waiting-list control group was matched to the desensitized group by focusing on nondesensitized patients having survived on the waiting list a time similar to the waiting time of desensitized patients. This prevents generalization to the whole cohort of patients registered on the waiting list. This difference in control groups between the one in the study by Orandi *et al.* ^9^ and the one in the study by Manook *et al.*^10^ and ours might explain the difference in the reported desensitization effects. Both of those studies are national registry studies whereas we report a single-center experience of desensitization. Despite the smaller population and the risk to miss a significant difference in survival, this study is a real-life study and all patients had the same desensitization protocol, which makes this cohort more homogeneous than other studies in this field. Our results provide further evidence that desensitization is not detrimental when compared to the full population of wait-listed patients and suggest a beneficial effect of desensitization.

Also, our data validate the use of a desensitization strategy for deceased-donor transplantations, an unprecedented result that opens up new possibilities for highly sensitized patients without a compatible living donor. Without desensitization strategies, these patients must wait for a deceased donor for a long period: they are often on hemodialysis and have increased morbidity/mortality.^8^ Despite the decision algorithm proposed by Keith *et al.*^20^, which integrated the paired-donation program, desensitization remains the only option for our patients due to a lack of efficiency in the paired-donation system in France. Our data argue in favor of desensitization, whether for a living or a deceased donor. The longer median time on a waiting list for deceased-donor transplant recipients (37 months) versus living-donor transplant recipients (14 months) might even lead to an overestimation of the death risk in our desensitization patients. The statistically significant association between DCGS and time spent on the waiting list confirmed here favors such an overestimation.

Pretransplantation DSAs increase the risks of ABMR and allograft loss.^21^^,^^22^ Yet these risks should be put into perspective with the risk of waiting for a compatible graft on a transplant list. In the literature, the benefit-risk balance and cost effectiveness when offering a desensitization protocol to highly sensitized patients remain uncertain.^23^

Our results are similar to those of Manook *et al.*^10^ as we could not show any survival advantage of desensitization. However, it did allow highly sensitized patients to receive a transplant and probably improved their quality of life. Eighty-three percent of the desensitized patients received a transplant within a maximum of 6 months, whereas only 51% of the nondesensitized patients were matched with a donor.

Regarding kidney function, in our experience, KT after desensitization had satisfactory results in terms of graft survival. In the literature, the rate of ABMR and graft loss is high.^24^ In our study, 36.6% of patients had an ABMR. In other studies, the rate of acute rejection was similar (approximately 36% of desensitized patients).^12^^,^^25^ Despite this increased risk, kidney graft survival of KTs was similar to that of HLA-compatible KTs. Mean estimated glomerular filtration rate at 1 year post-KT was similar in the groups of desensitized KT patients as compared to compatible KTs. However, the duration of follow-up in our study may not have been long enough and is significantly shorter in the desensitized KT group to determine whether desensitized patients had a greater risk of chronic ABMR and transplant glomerulopathy.^26^

We may suspect that DSA intensity before desensitization and DSA evolution over time may be correlated with the occurrence of rejection and long-term graft survival, yet these specific factors have yet to be investigated. Assessment of MFI alone is imperfect because there are several immunophenotypes of sensitization that are shaped by the source and duration of sensitization or the type and subclass of antibodies generated. Better assessment of the immunological risk will allow safer and more effective use of desensitization.^27^

In conclusion, desensitization is an effective strategy to achieve transplantation for highly sensitized patients. We assessed the impact of desensitization to facilitate KT access in highly sensitized patients and found similar survival rates for patients on the waiting list and for nondesensitized transplant recipients, while adjusting for the usually unaccounted immortal time bias. DCGS was also not different between desensitized and nondesensitized kidney transplant recipients, despite a higher risk of ABMR. This suggests further studies with a longer follow-up to provide longer-term outcome information.

This study has some limitations that may limit the generalization, such as the small size of the population which limits the precision of effect estimates, and the monocentric and retrospective nature of the study. Moreover, the desensitization protocol started in 2016, which limits the survival comparison with patients transplanted longer ago. Yet, less than 30% of patients were registered in the waiting list before 2010, and taking into account the immortal bias alleviates the impact because the time on waiting list for KT counts for the survival of patients on waiting list. Moreover, no patients in this cohort of highly sensitized patients underwent KT before 2009. Finally, desensitization gives to the underprivileged highly sensitized population the possibility of receiving a transplant with reassuring patient and graft survival results with the potential to improve quality of life.

## Disclosure

The authors declared no competing interests.

## Patient Consent

Informed consent was obtained from all subjects involved in the study.

## Supplementary Material

Supplementary File (PDF)

**Figure S1.** Flow chart.

**Figure S2.** Time-dependent Cox's survival model of desensitized patients and comparable highly sensitized patients. In this model, time spent on the wait-list counted as up to the time of transplantation for patients that eventually received a transplant. Survival curve of desensitized patients in the setting of HLA-incompatible kidney transplantation (light blue) is compared to highly sensitized patients remaining on the kidney-transplant waiting list (WL) at follow-up (dark blue) and to highly sensitized patients that received a transplant without desensitization (green). The at-risk number of patients varies over time in a nonlinear way in this model and cannot be calculated.

**Table S1.** Causes of death and graft loss at follow-up in the desensitized group.

## References

[1] Liyanage T., Ninomiya T., Jha V. (2015). Worldwide access to treatment for end-stage kidney disease: a systematic review. Lancet.

[2] Wolfe R.A., Ashby V.B., Milford E.L. (1999). Comparison of mortality in all patients on dialysis, patients on dialysis awaiting transplantation, and recipients of a first cadaveric transplant. N Engl J Med.

[3] Jackson K.R., Motter J.D., Kernodle A. (2020). How do highly sensitized patients get kidney transplants in the United States? Trends over the last decade. Am J Transplant.

[4] Pruthi R., Hilton R., Pankhurst L. (2013). UK Renal Registry 16th annual report: chapter 4 demography of patients waitlisted for renal transplantation in the UK: national and centre-specific analyses. Nephron Clin Pract.

[5] Claas F.H.J., Witvliet M.D., Duquesnoy R.J., Persijn G.G., Doxiadis I.I.N. (2004). The acceptable mismatch program as a fast tool for highly sensitized patients awaiting a cadaveric kidney transplantation: short waiting time and excellent graft outcome. Transplantation.

[6] Sethi S., Choi J., Toyoda M., Vo A., Peng A., Jordan S.C. (2017). Desensitization: overcoming the immunologic barriers to transplantation. J Immunol Res.

[7] Haririan A., Nogueira J., Kukuruga D. (2009). Positive cross-match living donor kidney transplantation: longer-term outcomes. Am J Transplant.

[8] Sapir-Pichhadze R., Tinckam K.J., Laupacis A., Logan A.G., Beyene J., Kim S.J. (2016). Immune sensitization and mortality in wait-listed kidney transplant candidates. J Am Soc Nephrol.

[9] Orandi B.J., Montgomery R.A., Segev D.L. (2016). Kidney transplants from HLA-incompatible live donors and survival. N Engl J Med.

[10] Manook M., Koeser L., Ahmed Z. (2017). Post-listing survival for highly sensitised patients on the UK kidney transplant waiting list: a matched cohort analysis. Lancet.

[11] Couzi L., Manook M., Perera R. (2015). Difference in outcomes after antibody-mediated rejection between abo-incompatible and positive cross-match transplantations. Transpl Int.

[12] Marfo K., Lu A., Ling M., Akalin E. (2011). Desensitization protocols and their outcome. Clin J Am Soc Nephrol.

[13] Clayton P.A., Coates P.T. (2017). Are sensitized patients better off with a desensitization transplant or waiting on dialysis?. Kidney Int.

[14] Montgomery R.A., Lonze B.E., King K.E. (2011). Desensitization in HLA-incompatible kidney recipients and survival. N Engl J Med.

[15] Gleiss A., Oberbauer R., Heinze G. (2018). An unjustified benefit: immortal time bias in the analysis of time-dependent events. Transpl Int.

[16] Suissa S. (2007). Immortal time bias in observational studies of drug effects. Pharmacoepidemiol Drug Saf.

[17] Zhou Z., Rahme E., Abrahamowicz M., Pilote L. (2005). Survival bias associated with time-to-treatment initiation in drug effectiveness evaluation: a comparison of methods. Am J Epidemiol.

[18] Kim S.J. (2010). Immortal time bias in cohort studies of kidney transplant recipients. Am J Transplant.

[19] Musoro J.Z., Struijk G.H., Geskus R.B., Ten Berge I., Zwinderman A.H. (2018). Dynamic prediction of recurrent events data by landmarking with application to a follow-up study of patients after kidney transplant. Stat Methods Med Res.

[20] Keith D.S., Vranic G.M. (2016). Approach to the highly sensitized kidney transplant candidate. Clin J Am Soc Nephrol.

[21] Lefaucheur C., Loupy A., Hill G.S. (2010). Preexisting donor-specific HLA antibodies predict outcome in kidney transplantation. J Am Soc Nephrol.

[22] Morath C., Opelz G., Zeier M., Süsal C. (2014). Clinical relevance of HLA antibody monitoring after kidney transplantation. J Immunol Res.

[23] Toyoda M., Shin B.-H., Ge S. (2017). Impact of desensitization on antiviral immunity in HLA-sensitized kidney transplant recipients. J Immunol Res.

[24] Niederhaus S.V., Muth B., Lorentzen D.F. (2011). Luminex-based desensitization protocols: the University of Wisconsin initial experience. Transplantation.

[25] Stegall M.D., Gloor J., Winters J.L., Moore S.B., Degoey S. (2006). A comparison of plasmapheresis versus high-dose IVIG desensitization in renal allograft recipients with high levels of donor specific alloantibody. Am J Transplant.

[26] Eskandary F., Bond G., Kozakowski N. (2017). Diagnostic contribution of donor-specific antibody characteristics to uncover late silent antibody-mediated rejection-results of a cross-sectional screening study. Transplantation.

[27] Zhang R. (2018). Donor-specific antibodies in kidney transplant recipients. Clin J Am Soc Nephrol.

