# Supplementary material for: Immortal Time-Bias–Corrected Survival of Highly Sensitized Patients and HLA-desensitized Kidney Transplant Recipients
Source: Kidney Int Rep. 2021 Aug 2;6(10):2629–38. doi: 10.1016/j.ekir.2021.07.024 (PMC8484495; doi:10.1016/j.ekir.2021.07.024)
Supplement: Supplementary File (PDF) [file mmc1.pdf]

Supplementary Figure 1. Flow chart

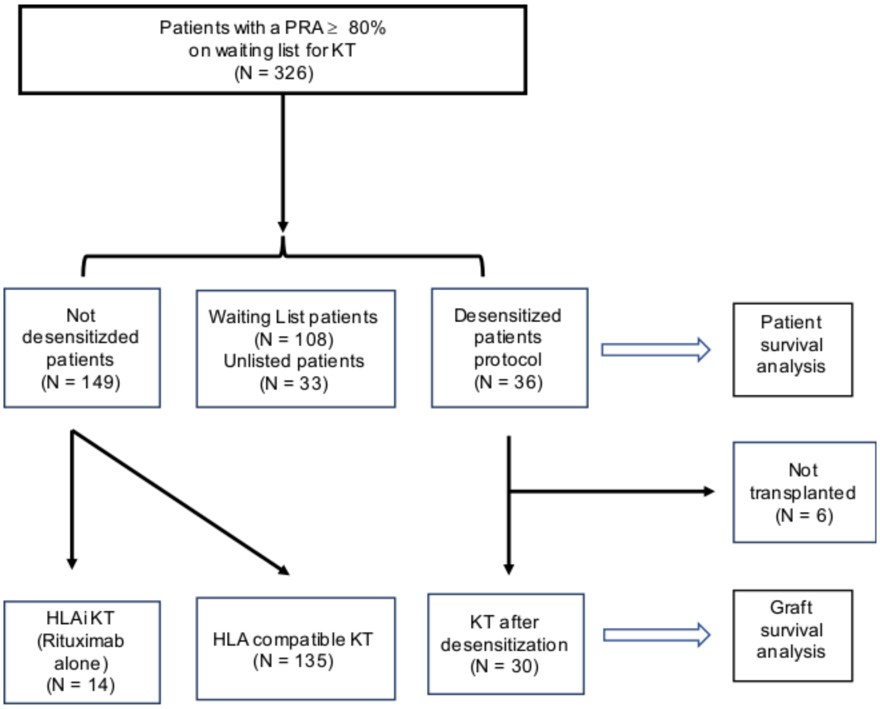

Supplementary Figure 2. Time-dependent Cox's survival model of desensitized patients and comparable highly sensitized patients. I

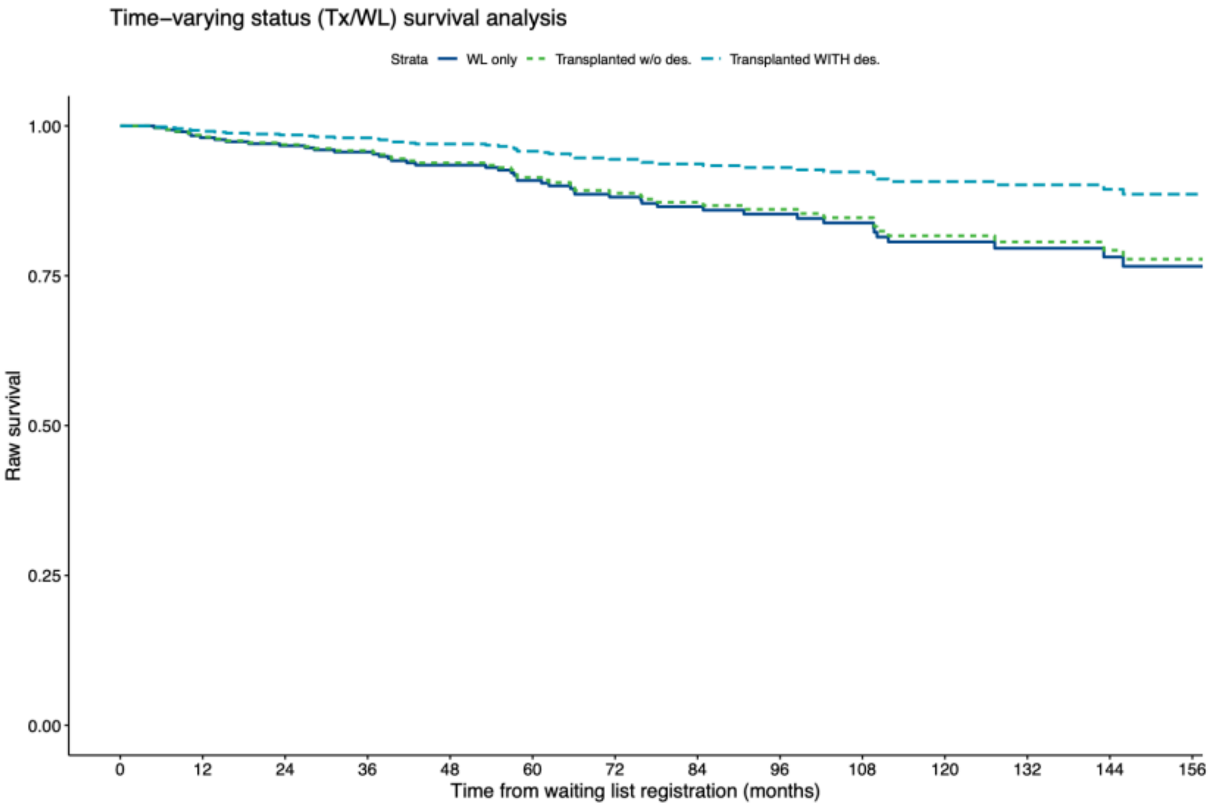

n this model, time spent on the wait-list counted as up to the time of transplantation for patients that eventually received a transplant. Survival curve of desensitized patients in the setting of HLA-incompatible kidney transplantation (light blue) is compared to highly sensitized patients remaining on the kidney-transplant waiting list (WL) at follow-up (dark blue) and to highly sensitized patients that received a transplant without desensitization (green). At risk number of patients varies over time in a non-linear way in this model and cannot be calculated.

Supplementary Table 1. Causes of death and graft loss at follow-up in the desensitized group

**Cause of death**	
Patient 1	Acute pulmonary edema on constricted aortic stenosis
Patient 2	Post-transplant multi-visceral failure complicated by digestive ischemia
Patient 3	Unknown cardiac arrest
Patient 4	Post-coronary artery bypass surgery complicated by multi-visceral failure and digestive ischemia
**Cause of graft loss**	
Patient 5	Acute tubular necrosis with no recovery of primary function
Patient 6	Antibody mediated rejection (DSA+)
Patient 7	Bacterial infection of the postoperative site with rupture of the arterial anastomosis
Patient 8	Antibody mediated rejection (DSA+)
Patient 9	Chronic active antibody mediated rejection (DSA+)

DSA: donor-specific antibody
